# Anesthetic Considerations for Pulmonary Arteriovenous Malformation Embolization in a Patient With Hereditary Hemorrhagic Telangiectasia: A Case Report

**DOI:** 10.7759/cureus.111545

**Published:** 2026-06-26

**Authors:** Hudson Martins de Brito, Camila Funatsu Magalhaes, Arjun Haresh Ghodasara, Livia Dahmen Rodrigues, Tiago de Almeida Macruz

**Affiliations:** 1 Department of Medicine, Federal University of Ceará, Fortaleza, BRA; 2 Department of Anesthesiology, University of Miami Miller School of Medicine, Jackson Memorial Hospital, Miami, USA; 3 Department of Medicine, University of Miami Miller School of Medicine, Miami, USA; 4 Department of Anesthesiology, University of Miami, Miami, USA

**Keywords:** anesthetic management, hereditary hemorrhagic telangiectasia (hht), ​perioperative care, pulmonary arteriovenous malformations, transcatheter embolization

## Abstract

Hereditary hemorrhagic telangiectasia (HHT) is characterized by mucocutaneous telangiectases and visceral arteriovenous malformations (AVMs). Pulmonary AVMs (PAVMs) create an anatomic right-to-left shunt, increasing the risk of hypoxemia and paradoxical embolic events, and are commonly treated with transcatheter embolization. We report a 25-year-old man with suspected HHT based on Curaçao criteria, including recurrent epistaxis, prior cerebral AVM, and multiple PAVMs, presenting with exertional dyspnea and hypoxemia. He underwent embolization under general anesthesia with air management, lung isolation capability, and tailored ventilatory management. The procedure was completed without complications, with resolution of symptoms and normalization of oxygenation. This case highlights the importance of understanding PAVM pathophysiology and implementing targeted anesthetic strategies to ensure safe perioperative management.

## Introduction

Hereditary hemorrhagic telangiectasia (HHT) is a vascular disorder characterized by mucocutaneous telangiectases and visceral arteriovenous malformations (AVMs), which may result in recurrent bleeding and vascular shunting [1]. It follows an autosomal dominant inheritance pattern and is caused by loss-of-function variants affecting members of the transforming growth factor-beta (TGF-β)/bone morphogenetic protein (BMP) signaling pathway [2]. Clinically, the diagnosis is established when at least three of the four criteria are met, and includes recurrent epistaxis, mucocutaneous telangiectasis, visceral involvement, and positive family history [1,2].

Pulmonary and hepatic AVMs affect about one in two people with HHT and are the major drivers of hemodynamic instability and perioperative risk [2]. Systemic AVMs promote left-to-right shunting, predisposing to high-output cardiac failure. In contrast, pulmonary AVMs create an anatomic right-to-left shunt, allowing blood to bypass the pulmonary capillary bed and thus the alveolar gas exchange. This mechanism increases susceptibility to hypoxemia and paradoxical embolic events, including ischemic strokes and brain abscesses [1,2]. In addition, pulmonary AVMs carry a risk of rupture, which may lead to potentially fatal hemoptysis or hemothorax.

Embolization is recommended even for asymptomatic patients with radiographically detectable pulmonary AVMs (PAVMs), as it significantly reduces the risk from their complications [3,4]. This endovascular intervention is typically performed via venous access (femoral or jugular), and strict precautions must be taken to prevent air entrainment, given the risk of catastrophic embolic events [5].

The anesthetic management of patients with HHT is particularly challenging, as disease-related complications may arise throughout the perioperative care. Key considerations include meticulous elimination of intravenous air (with consideration of in-line filters), strategies to mitigate the effects of right-to-left shunting, preparedness for lung isolation in the event of intrapulmonary hemorrhage, and careful avoidance of nasal or oropharyngeal instrumentation due to the presence of fragile mucosal telangiectases [6].

We report the case of a patient with suspected HHT based on Curaçao criteria and multiple pulmonary arteriovenous malformations undergoing transcatheter embolization in the interventional radiology suite, managed according to current standards of anesthetic care. Written informed consent for the publication of this case report was obtained.

## Case presentation

A 25-year-old man presented with three days of dyspnea and intermittent chest discomfort. His medical history was notable for brain surgery at the age of five and a lifelong history of frequent epistaxis. His mother reported a similar bleeding history. On presentation, the patient was mildly hypoxemic, with oxygen saturation of 88%-92% on room air, but improved with oxygen administration via nasal cannula. He also had a previously documented history of unexplained exertional desaturation.

The chest computed tomography (CT) revealed three pulmonary AVMs (Figure 1). Two adjacent lesions in the right lower lobe (1.5-1.8 cm) and a similar lesion in the left lower lobe, but no pulmonary embolism was seen. Laboratory studies demonstrated persistently elevated hemoglobin levels ranging from 16.2 to 17.9 g/dL (Figure 2, M1-M3), consistent with chronic hypoxemia. Transthoracic echocardiogram (TTE) demonstrated normal biventricular function without evidence of pulmonary hypertension (Figure 3).

**Figure 1:**
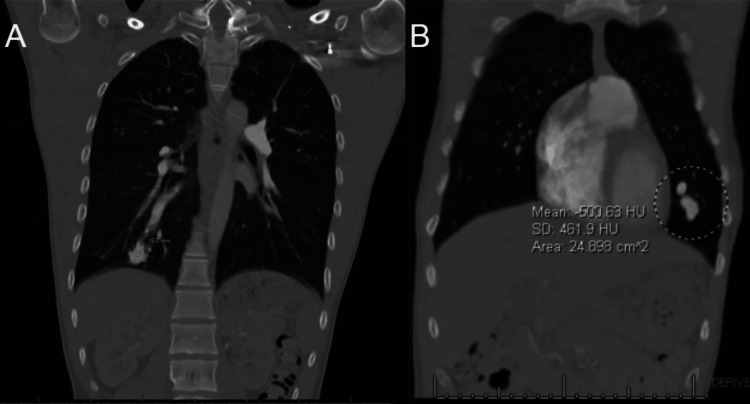
Computed tomography angiography prior to the procedure, demonstrating an AVM in the right and left lobes. (A) Computed tomography angiography prior procedure, demonstrating an AVM measuring approximately 18 mm in the right lower lobe; (B) computed tomography angiography prior procedure, demonstrating an AVM in the left lower lobe. AVM: arteriovenous malformation.

**Figure 2:**
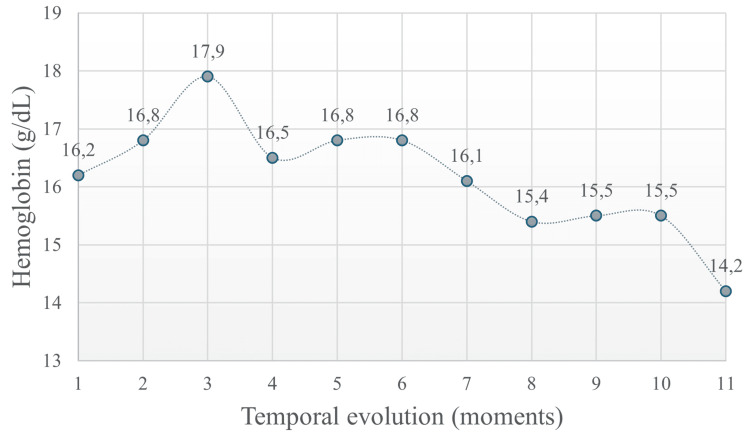
Temporal evolution of hemoglobin levels following staged embolization of pulmonary arteriovenous malformations (AVMs). Hemoglobin reference range (male): 13 to 18 g/dL. Hemoglobin levels were obtained at 11 time points throughout the patient's follow-up. M1 and M2 correspond to baseline assessments before intervention. M3 marks the first embolization procedure, during which two AVMs in the right lower lobe were treated. M4 through M7 represent measurements obtained during the post-procedural hospitalization and at discharge. M8 and M9 correspond to evaluations performed four months later, prior to the second embolization. M10 marks the second embolization procedure, targeting the remaining AVM in the left lower lobe. M11 represents the follow-up assessment 42 days after the second procedure. A progressive decline in hemoglobin levels was observed following the embolizations, with normalization after complete treatment of the pulmonary AVMs.

**Figure 3:**
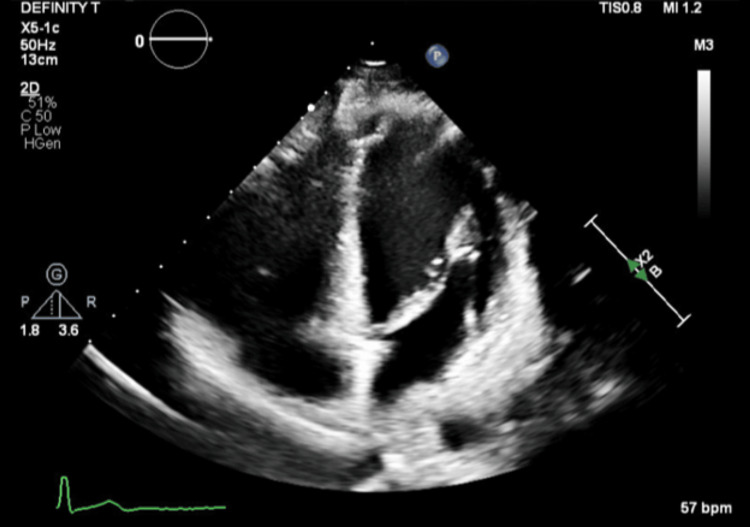
Transthoracic echocardiogram (TTE) demonstrating normal cardiac chamber dimensions and preserved biventricular function. No echocardiographic evidence of pulmonary hypertension was identified.

Neuroimaging, including computed tomography angiography (CTA; Figure 4A), magnetic resonance imaging (MRI; Figure 4B), and magnetic resonance angiography (MRA; Figure 4C), revealed embolization material in the left temporal lobe with surrounding encephalomalacia consistent with a treated childhood AVM. The presence of prior cerebral AVM, recurrent epistaxis, and new pulmonary AVMs met ≥2 Curaçao criteria [7], supporting the diagnosis of HHT.

**Figure 4:**
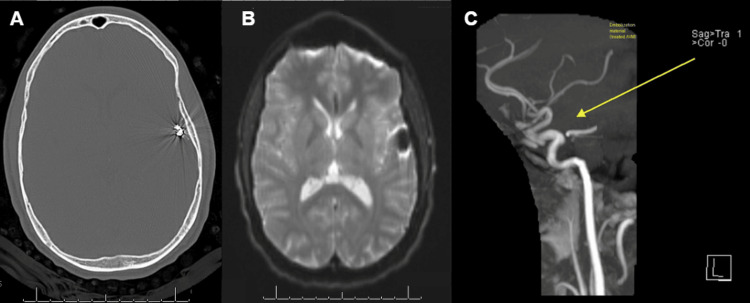
Neuroimaging reveals embolization material in the left temporal lobe associated with surrounding encephalomalacia, compatible with previous embolization of a childhood arteriovenous malformation (AVM). (A) Computed tomography angiography (CTA); (B) magnetic resonance imaging (MRI); (C) magnetic resonance angiography (MRA).

Given the risk of hemorrhage from pulmonary AVMs and potential need for lung isolation, general anesthesia was induced with arterial line placement and meticulous avoidance of air in all IV systems. A 35 Fr left-sided double-lumen tube (Parker Medical, Highlands Ranch, CO, USA) was placed and confirmed via bronchoscopy to enable rapid one-lung ventilation in case of bleeding. Ventilatory management targeted normocapnia, low airway pressures, and high FiO₂. Nitrous oxide was avoided.

During the interventional radiology procedure, femoral venous access and all catheter exchanges were performed under continuous saline infusion to prevent air entrainment. Angiography identified large AVMs in the right middle and right lower lobes with multiple aneurysmal sacs, and these were successfully embolized with coils. A small lingular AVM could not be accessed.

The procedure was completed without complications, with no episodes of desaturation, hemodynamic instability, or pulmonary hemorrhage (Figures 5, 6). The patient had an uneventful recovery and was discharged on postoperative day one. At discharge, he was asymptomatic, with resolution of dyspnea and oxygen saturation maintained at 99% on room air. 

**Figure 5:**
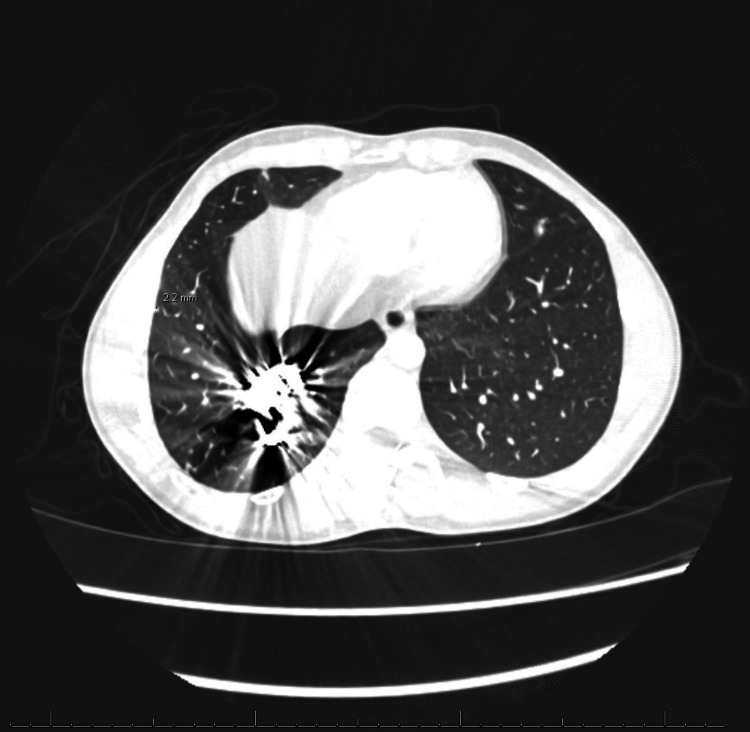
Post-procedure computed tomography after the first embolization in the right lower lobe.

**Figure 6:**
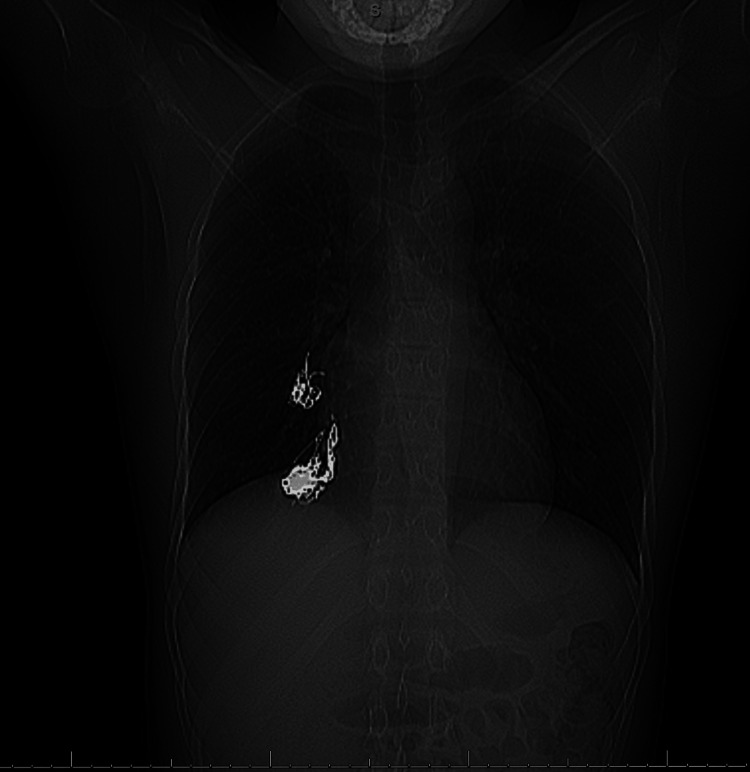
Post-procedure computed tomography after the first embolization in the right lower lobe.

Four months later, a second embolization procedure was performed to treat the remaining pulmonary AVM using the same anesthetic and interventional approach. Following embolization of the first two AVMs, the patient demonstrated a reduction in hemoglobin levels with normalization toward the reference range. Forty-two days after the second procedure, hemoglobin levels decreased further and remained within normal limits (Figure 2, M11), suggesting resolution of the chronic hypoxemia-induced secondary erythrocytosis.

## Discussion

Hereditary hemorrhagic telangiectasia (HHT) is a multisystem vascular disorder resulting from abnormal angiogenesis related to mutations affecting the TGF-β/BMP signaling pathway, most commonly involving the ENG and ACVRL1 genes [2]. These alterations result in fragile mucocutaneous telangiectases and visceral arteriovenous malformations (AVMs), characterized by direct arterial-venous communications without an intervening capillary bed [1].

Clinically, the diagnosis relies on the Curaçao criteria [7], which include recurrent epistaxis, mucocutaneous telangiectases, visceral AVMs, and a compatible family history. The presence of three of these findings establishes a definite diagnosis [1,2]. In the present case, the combination of lifelong epistaxis, prior cerebral AVM, and newly identified pulmonary AVMs strongly suggested the diagnosis of HHT. Recognition of this constellation is particularly relevant in the perioperative setting, as visceral AVMs may significantly influence anesthetic risk and procedural planning.

This case describes the anesthetic management of a young patient with suspected HHT and multiple PAVMs undergoing transcatheter embolization in the interventional radiology suite. The pathophysiological consequences of PAVMs are primarily driven by a fixed right-to-left shunt, which allows a portion of venous blood to bypass the pulmonary capillary filtration system and thus the alveolar gas exchange [1,2]. This results in reduced arterial oxygenation and increased susceptibility to paradoxical embolic events, including ischemic stroke and brain abscesses [1].

Chronic hypoxemia is common and may be exacerbated by exertion, while compensatory erythrocytosis partially preserves oxygen delivery. In our patient, this was reflected by chronically elevated hemoglobin levels (16.2 to 17.9 g/dL g/dL) in the absence of pulmonary hypertension or obstructive lung disease. Additionally, PAVMs carry a risk of rupture, potentially leading to severe and fatal hemoptysis or hemothorax.

The coexistence of fixed right-to-left shunting, chronic hypoxemia, and the risk of intrapulmonary hemorrhage creates unique perioperative challenges. Hypoxic pulmonary vasoconstriction (HPV) is a physiological reflex that redirects pulmonary blood flow away from poorly ventilated lung regions to optimize ventilation-perfusion matching [8]. However, this protective mechanism is ineffective, and potentially counterproductive, in the setting of PAVMs [8]. Unlike functional shunts caused by atelectasis or consolidation, PAVMs represent fixed anatomical communications that lack the smooth muscle layer necessary to respond to hypoxic stimuli. Consequently, when systemic or alveolar hypoxia triggers generalized HPV in the surrounding normal vasculature, pulmonary artery pressure rises globally, while the resistance across the PAVM remains unchanged [9].

Applying the fundamental flow equation (Q = ΔP/R), an increase in upstream pulmonary arterial pressure with a fixed low-resistance pathway inevitably results in increased shunt flow through the malformation. This creates a self-reinforcing cycle where hypoxia induces HPV, elevating pulmonary artery pressure, augmenting flow through the PAVM, and worsening arterial hypoxemia [8,9].

Interestingly, general anesthesia introduces a complex interaction with this physiology that may paradoxically confer a degree of protection. Volatile anesthetic agents are well-established inhibitors of HPV in a dose-dependent fashion, and intravenous agents such as propofol also attenuate this reflex to a variable degree [8]. By blunting HPV in the normal pulmonary vasculature, these agents reduce the generalized rise in pulmonary artery pressure that would otherwise augment shunt flow. This stands in contrast to functional shunts, such as those caused by atelectasis, where HPV inhibition is unequivocally detrimental [8].

This protective window, however, is contingent on avoiding conditions that restore or exceed baseline pulmonary artery pressure, including hypercarbia, excessive positive end-expiratory pressure (PEEP), and hypoxia [10]. Therefore, our ventilatory management strategy targeted at normocapnia and low airway pressures, minimizing any stimulus for pulmonary vasoconstriction that could re-engage the paradoxical shunt-augmenting loop. In our case, placement of a double-lumen tube was placed to allow immediate one-lung ventilation in the event of sudden intrapulmonary bleeding. This precaution is vital because airway management in patients with HHT requires extreme caution due to fragile mucosal telangiectases and the risk of severe pulmonary hemorrhage [6].

Given these risks, transcatheter embolization is considered the standard treatment for radiographically detectable PAVMs, even in asymptomatic individuals, as it reduced the incidence of embolic and hemorrhagic complications while improving oxygenation [3,4]. The procedure is typically performed in the interventional radiology setting via femoral venous access. Despite being minimally invasive, strict precautions are required to prevent air entrainment during catheter manipulation, as even small volumes of gas may result in paradoxical embolism [5].

In our patient, meticulous measures were taken to eliminate air from all intravenous systems, and catheter manipulations were performed under continuous saline infusion in close coordination with the interventional radiology team. Nitrous oxide was avoided due to its potential to expand intravascular gas and increase pulmonary resistance [11], factors that may contribute to an increased risk of embolic complications. It's important to emphasize that no neurologic or hemodynamic complications occurred during the procedure or were observed in the following days.

Furthermore, the hemodynamic management of these patients with PAVMs under general anesthesia requires careful consideration of the differential effects of vasopressors on systemic (SVR) and pulmonary vascular resistance (PVR). Anesthesia-induced vasodilation frequently necessitates vasopressor support, but the choice of agent carries physiological implications that are amplified in the presence of a fixed intrapulmonary shunt. Phenylephrine, a pure alpha-1 agonist, increases SVR but also raises PVR, potentially augmenting shunt flow through the low-resistance PAVM pathway [12]. Vasopressin, by contrast, exerts potent systemic vasoconstriction through V1 receptors while having a comparatively neutral or even favorable effect on PVR, making it a theoretically preferable agent [13,14]. Norepinephrine occupies an intermediate position, with mixed alpha and beta-adrenergic activity that generally produces less pulmonary vasoconstriction than phenylephrine at equipotent systemic doses [12].

Beyond vasopressor selection, nitrous oxide warrants particular mention as a dual contraindication in patients with PAVMs [11]. In addition to its well-recognized risk of expanding intravascular gas bubbles and potentiating paradoxical embolism, nitrous oxide may also increase pulmonary vascular resistance [11]. These effects could potentially alter shunt dynamics and contribute to worsening arterial oxygenation, providing further rationale for avoiding nitrous oxide in this patient population.

Overall, this case highlights the importance of understanding the pathophysiology of PAVMs and its implications for anesthetic management. Prevention of air embolism, careful airway planning, and coordination between anesthesiology and interventional radiology teams are essential to ensure procedural safety. With appropriate preparation and adherence to those principles, transcatheter embolization of PAVMs can be performed safely in patients with suspected or confirmed HHT.

## Conclusions

Patients with hereditary hemorrhagic telangiectasia and pulmonary arteriovenous malformations present unique challenges for anesthetic management due to right-to-left shunting, chronic hypoxemia, and the risk of paradoxical embolization and pulmonary hemorrhage. Successful perioperative care requires careful planning, including strict prevention of intravenous air entry, ventilatory strategies that avoid increases in pulmonary vascular resistance, and preparedness for rapid lung isolation if intrapulmonary bleeding occurs. With these precautions and close coordination between anesthesiology and interventional radiology teams, transcatheter embolization of pulmonary AVMs can be performed safely and effectively.
